# A Case of Psoas Abscess Successfully Treated

**Published:** 1793

**Authors:** William Smith

**Affiliations:** Surgeon at Bideford, and Member of the Corporation of Surgeons of London.


XII. *A Case of Psoas Abscess successfully treated. By Mr.* William Smith, *Surgeon at Bideford, and Member of the Corporation of Surgeons of London. Communicated in a Letter to* Edward Whitaker Gray, *M. D. F. R. S.; and by him to Dr.* Simmons.

A YOUNG lady of this town, aged eighteen years, of a fair, ruddy complexion, and rather inclined to plumpness, was attacked whilst she was riding (thinly cloathed) over a bleak common, about the middle of December, 1787, with pain in her back, but was not prevented by it from pursuing her journey, and spending several days with her friends.

She returned to Bideford on the 30th of the same month, when I was first requested to visit her. She complained of a violent and continued pain across her back and loins, which had gradually increased from the time above mentioned, attended with shooting pains through the right groin, in the direction of the psoas muscle, and extending down the thigh; the erect posture always produced increase of pain, and every attempt to roll the thigh bone in the acetabulum (when

(when I was endeavoring to detect the immediate seat of the disease) occasioned the most excruciating torture. Her cheeks were flushed, her tongue white and dry, with an insatiable thirst, her pulse hard and full, in number 120, no stools had been procured for several days, and the urine was voided in small quantities, and very high coloured.

Judging from these symptoms that inflammation had taken place in that portion of the cellular membrane which is immediately connected with the psoas and iliacus internus muscles, and, being in hopes that the suppurative stage had not yet begun, I deemed it warrantable to endeavour to effect the cure by resolution.

I ordered her to be put to bed, with a firm injunction to preserve the position recommended, which was laying on her side, with the thigh in a right angle with the trunk, in order to shorten, as much as possible, the space between the origin and insertion of the psoas muscle. Blood was taken from a large orifice in the arm, *ad deliquium*, copious stools were procured by means of purgative medicines and enemas, and a large blister was applied over the lumbar vertebræ; she took an antimonial every six hours, with small doses of tincture of opium; and a diluent farinaceous rinaceous diet was ſtrictly enjoined, with a total prohibition of all fermented liquors and animal food.

Notwithſtanding this mode of treatment, which ſhe ſubmitted to with great perſeverance, the ſymptomatic fever rather increaſed than diminiſhed; every attempt to move her in bed produced agony; and a perpetual anxiety and watchfulneſs rendered her ſituation truly deplorable.

On the 7th of January, 1788, a ſevere rigor took place, which was followed by ſeveral others during the three ſucceeding days.

The violence of her pains began now ſenſibly to diminiſh; her pulſe became ſofter; partial remiſſions of fever enſued; ſleep was procured by means of opium, and ſhe experienced temporary ſuſpenſion of her miſery.

Revolving in my mind the foregoing ſymptoms, I had no doubt remaining (although no external tumor yet appeared) of the exiſtence of ſuppuration, and I became every day more and more ſolicitous to examine the lumbar region and inguina, being determined to give vent to the matter as ſoon as it came within reach of the knife; this, however, from too great delicacy, was conſtantly denied me.

From

From the 7th to the 20th it was obvious that my patient loſt ſtrength daily; although the acuteneſs of the pain had ſubſided, the ſymptomatic fever continued with fewer remiſſions, profuſe perſpirations came on, with conſiderable emaciation, and the legs and thighs became anaſarcous. Her pulſe was now extremely frequent, ſmall, and tremulous, varying in number, from 120 to 140, upon the ſlighteſt agitation or exertion of body.

Wine, Broths, and Jellies, were now added to her diet, with the free uſe of cow's milk; and the maturation of the abſceſs was forwarded by warm fomentations: ſtill, however, the nurſe aſſured me (for I had ordered her to make frequent examination,) that no particular hardneſs or ſwelling appeared.

On the 29th of January, ſhe was viſited by Dr. Wavell, of Barnſtaple, at my requeſt. He confirmed me in my opinion of the exiſtence of internal ſuppuration, encouraged me in the determination of opening it, as ſoon as the ſmalleſt tumor ſhould indicate the direction it would take, and conſented to be preſent at the operation; in the mean time preſcribing for her the bark, with the occaſional uſe of opium.

On

On the 4th day of February, I was called to her in the evening; the nurſe informed me that ſhe complained of a ſwelling in the right groin, which occaſioned great pain in turning, and that ſhe no longer oppoſed an examination.

I found the tumor occupying the whole of the right groin, with evident fluctuation; the tenſeneſs of it increaſed upon raiſing her to the erect poſture, but it became flaccid upon laying her ſupine, and the whole contents might with eaſe be preſſed into the cavity of the abdomen: the ſhape of the tumor was a flattened pyriform; its greateſt length was from the ſpine of the ilium to the pubis; and there was little or no diſcoloration.

On the 5th of February, in the morning, in the preſence of Dr. Wavell, I opened the tumor, by making a puncture with a double-edged biſtoury below and in the direction of Poupart's ligament, and gave vent to a large quantity of brown, ichorous matter, the ſtench of which was ſo intolerably offenſive, that with difficulty we could remain in the room whilſt the aſſiſtants were collecting it in baſons, napkins, &c; and, incredible as it may appear, between thirty and forty of the latter were completely ſoaked in the firſt few hours after the operation. The deliquium deliquium induced by this ſudden and great depletion was ſuch, that I was under the neceſſity of ordering an aſſiſtant to preſs, at intervals, upon the opening, until the patient's ſtrength was ſufficiently recruited by wine and volatiles to bear a freſh diſcharge. Large portions of black, putrid, cellular ſubſtance floated through the aperture, and the firſt day elapſed before any kind of dreſſing could be retained, as the ſlighteſt motions of the body threw off conſiderable quantities of the ſame membranous ſloughs and fetid ſanies.

Dr. Wavell preſcribed for her half a drachm of peruvian bark in a ſtrong decoction of the ſame every five hours, with one grain of opium; and ordered ſtools to be procured by occaſional enemas.

The room was well ventilated, and the putrid effluvia counteracted, as much as poſſible, by antiſeptic fumigations of camphor, vinegar, &c.

In a few days after the operation, her appetite for food returned, and conceiving our firſt ſtep was to adopt a nouriſhment proportionate to the daily waſte, I determined to arrange the order of her diet, and carefully obſerve the quantum of food ſhe could take in a day.

Her

Her diet confifted of boiled chickens, hartf-horn and calves-feet jellies, milk, foups, and blanc-mange, with the free ufe of port wine.

During the violence of the difcharge, I found her ftomach would bear very well two quarts of milk and a bottle of port wine every day, befides her dining on chickens, and taking every two or three hours a cup of jelly, &c.

For the firft three weeks the difcharge continued profufe, thin, and putrid, excoriating the lips of the aperture, and bringing along with it confiderable portions of floughy cellular membrane, which fometimes choaked up the opening and obftructed the drain: her pulfe was feldom under 120 in number, fmall, and hard; a conftant anxiety prevented fleep, and the emaciation of the arms and trunk became every day more vifible, whilft the lower limbs were bloated with anafarca to the rim of the belly. Obferving that every elevation of body poured out a torrent of fanies, I ordered her to be gently raifed on pillows, inclining to the difeafed fide, in order to obtain a depending orifice from the fac, and thereby effect a more regular difcharge.

By the firft week in March the difcharge was fomewhat abated, and the matter become of a thicker confiftence, of a paler brown colour, and and the ſmell was leſs offenſive; the ſymptomatic fever alſo was diminiſhed; her pulſe (in number 100) was more ſoft and open, ſleep was obtained by ſmall doſes of opium, and I began to conceive hopes that the caſe might terminate favourably.

Notwithſtanding theſe encouraging appearances, the dropſy of the lower limbs remained, the cuticle burſt in ſeveral places, and a quantity of lymph oozed out. Suſpecting the continuance of this troubleſome ſymptom to be the effect of mere local debility, (the ſkin having, from long preternatural diſtention, loſt its contractile power,) I made a gradual preſſure with flannel rollers, beginning at the feet, and finding I gained ground on the complaint thereby, I increaſed the preſſure every day, and by that mean gradually removed the whole œdema.

By the 25th of March the quality of the diſcharge was much improved; the fætor was gone, and it had a true purulent conſiſtence, leſſening in quantity every day: her pulſe became natural, and her tongue clean; her ſleep was undiſturbed and refreſhing, without the aid of opiates; the alvine diſcharges required little aſſiſtance from laxatives, and the catamenia, which had been obſtructed from the firſt attack, again began to flow.

On the 26th ſhe complained of great pain in her thigh, on the external part, a little below the great trochanter. Upon examination, a fluctuation (though deep ſeated) beneath the faſcia was manifeſt; and ſuſpecting (as the wound in the groin had diſcharged very little for ſeveral days) that the matter had made its way through the interſtices of the muſcles, I made a ſmall inciſion, which gave vent to about ſix ounces of pure pus, and having obtained thereby an orifice ſtill more dependent, the one in the groin ſoon cloſed.

She gained ſtrength now very rapidly; the anaſarca was gone; the fever, and its concomitant ſymptoms, had alſo left her; the wound of the groin was cloſed; and a ſmall aperture in the thigh diſcharged about an ounce of purulent matter at every dreſſing.

Being apprehenſive, however, that a long ſinus might yet remain, and that the delicate union of the cloſed parts might again be ſeparated by any ſudden turning or motion of the body, I ſtill enjoined the moſt abſolute reſt.

On the 10th of April, I perceived a crimſon ſuffuſion on the ſkin round the laſt opening, extending extending to the rim of the belly, and conceiving that an adhefive inflammation had taken place, which (if unmolefted) would unite the fides of the remaining finus, I withdrew a fmall doffil, and covered the wound fuperficially with lint.

My conjecture proved true, for in two or three days the difcharge totally ceafed, fhe gradually recovered her ftrength and blooming complexion, has enjoyed an uninterrupted ftate of health ever fince, and is totally free from lamenefs.

The exciting caufe in this inftance feems evidently to have been expofure to cold; and the happy termination of the difeafe proves that fuccefs may be expected fometimes to attend thefe deplorable cafes, which are too often (particularly in hofpitals) given up as abfolutely incurable.

During the progrefs of this cafe I always kept the following confiderations in view:

1. To prevent, as much as poffible, the admiffion of external air into the cavities, by operating by puncture only.

2. To appeafe pain and irritation, by a liberal uſe of opium, and the moſt ſimple ſuperficial dreſſings.

3. To endeavour to proportionate the quantum of nouriſhing diet and tonic medicines to the daily waſte by ſuppuration.

And, 4. To ventilate the room frequently, by paſſing currents of pure air through it; for which the ſituation of the houſe was peculiarly favourable, being by the river ſide.

*Bideford*,

Feb. 4, 1793.

